# Preventative approaches to falls and frailty

**DOI:** 10.1016/j.afos.2025.04.002

**Published:** 2025-05-09

**Authors:** Seiji Hashimoto, Tatsuya Hosoi, Mitsutaka Yakabe, Makoto Yunoki, Shoya Matsumoto, Yoshitaka Kase, Masashi Miyawaki, Masaki Ishii, Sumito Ogawa

**Affiliations:** Department of Geriatric Medicine, Graduate School of Medicine, The University of Tokyo, Tokyo, Japan

**Keywords:** Fall, Frailty, Sarcopenia, Comprehensive geriatric assessment, Geriatric syndrome

## Abstract

With increasing life expectancy, the aging population grows, leading to more age-related diseases. Among these, falls are a serious public health issue, as they cause disability in older adults and impose a significant burden both on caregivers and society. Despite numerous studies on fall risk and prevention, the incidence of falls among older adults has not shown any significant improvement. Given the multifactorial nature of falls, it is necessary to evaluate risk factors comprehensively. Recently, the Comprehensive Geriatric Assessment, a tool for comprehensively evaluating older adults, has been reported to help prevent falls and improve postfall outcomes. Assessing individual risks and effectively integrating exercise, nutrition, and appropriate medication management into multifactorial interventions is essential for developing effective fall prevention strategies.

## 1 Introduction

As people live longer, the number of older adults increases, raising the prevalence of geriatric syndromes. These syndromes often result in a decline in activities of daily living (ADL), requiring caregiving and support alongside medical interventions. Falls and fall-related injuries, together with frailty and sarcopenia, are particularly common and serious issues, posing critical public health challenges. Thus, their prevention is a healthcare priority for our aging society [1]. The multifaceted factors contributing to falls require a comprehensive evaluation and individualized interventions. This review provides an overview of the associations between frailty, sarcopenia, and falls and examines the effective interventions to prevent falls.

### 1.1 Falls in older adults

A fall is defined by the World Health Organization as “An event which results in a person coming to rest inadvertently on the ground or floor or other lower level. Falls, trips and slips can occur on one level or from a height.” They are typically due to underlying physical illnesses, medication, and environmental hazards, often combined [2,3]. In older adults, falls are a major cause of fractures and head injuries, leading not only to a decline in ADL but also to mortality [4]. Despite numerous studies on fall risk and prevention in older adults, as well as various existing fall prevention strategies [[5], [6], [7], [8]], the fall incidence among older adults has not improved significantly. Reportedly, 27.5% of people aged ≥ 65 experience a fall at least once annually, and 10.2% suffer injuries from falls [9]. Additionally, the fall-related mortality rate for people aged ≥ 75 has increased from 51.6 per 100,000 in 2000 to 122.2 per 100,000 in 2016 [10].

With declining sensory and motor functions, older adults often struggle to make rapid positional adjustments. In addition, highly prevalent comorbid conditions in this population, such as diabetes and cognitive impairment, further increase the risk of falls. Besides, this multimorbidity often leads to polypharmacy in older adults. In fact, fall risk-increasing drugs (FRIDs)—including diuretics, antihypertensive medications, psychotropics, and hypnotic drugs—elevate the likelihood of falls [[11], [12], [13]]. Notably, even within the same drug class, the fall risk can differ based on the specific medication used. For example, while hypnotic drugs are generally associated with an increased risk of falls and fractures, orexin receptor antagonists may not increase the risk of fractures [14].

Nearly 80% of falls among older adults occur indoors, with bedrooms, stairs, and bathrooms identified as primary locations. In addition, 31.6% of all indoor falls among individuals aged ≥ 85 occur in the bedroom. This may be partly attributed to the significant amount of time older adults spend in their bedrooms [15]. Additionally, the potential impact of hypnotic drugs, commonly prescribed for older adults, should not be overlooked, as their use may influence fall risk. Therefore, healthcare professionals involved in geriatric care must conduct comprehensive assessments of older adults and carefully select medications, including hypnotics, to ensure appropriate and safe treatment [14,16]. In addition, recent research has reported the potential of computer vision and artificial intelligence for fall detection; technologies which may contribute to fall prevention efforts in the future [17].

### 1.2 Association between falls, frailty, and sarcopenia

In older adults, falls result from the complex interplay of various factors, broadly categorized into internal and external factors [18,19]. Internal factors included frailty, sarcopenia, cognitive impairment, and gait abnormalities. External factors include the home environment, footwear, and walking aids. Among all, frailty and sarcopenia are significant risk factors for falls in older adults [20,21].

Frailty is defined as a state of increased vulnerability to stress due to age-related functional decline and loss of reserve capacity [22]. It encompasses not only physical frailty but also psychological and social frailty. While physical frailty is widely recognized as a major risk factor for falls, emerging evidence suggests that psychological and social frailty also contributes to fall risk [23,24]. Importantly, frailty is considered a reversible condition, and appropriate interventions could prevent the need for long-term care.

**Fig. 1:**
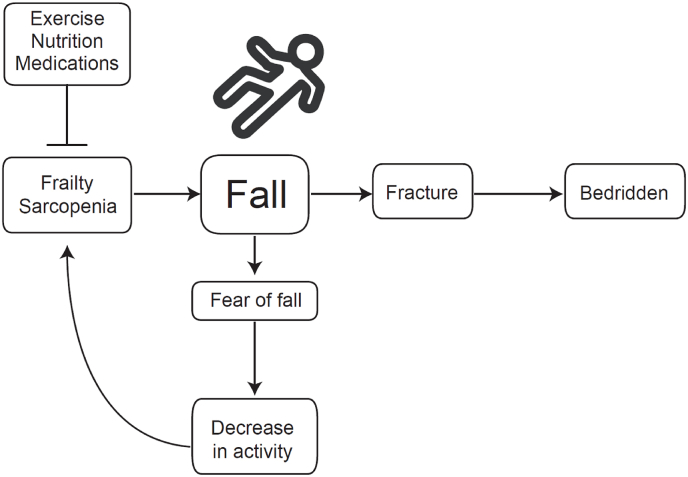
Conceptual model illustrating the vicious cycle of frailty, sarcopenia, falls, and fear of falling. Frailty and sarcopenia increase the risk of falls and fractures, leading to disability. Fear of falling reduces physical activity, which in turn exacerbates frailty and sarcopenia.

Sarcopenia refers to a decline in skeletal muscle mass accompanied by a loss of muscle strength or physical function in old age [25]. It has been officially recognized as a disease since 2016. Notably, even falls that result in no physical injury often have social and psychological consequences, including loss of confidence, mobility restrictions, and a significant prevalence of fear of falling [3,26]. As illustrated in Fig. 1, a bidirectional vicious cycle emerges in which the fear of falling leads to reduced activity levels, further worsening frailty and sarcopenia. Therefore, the prevention of both falls and sarcopenia is crucial. Frailty has recently been recognized as a state of disrupted homeostasis due to impaired interactions across multiple domains—genetic, biological, functional, cognitive, psychological, and socio-economic [27]. Such complexity can be systematically captured by comprehensive geriatric assessment (CGA), which offers a practical framework for evaluating frailty and guiding individualized fall prevention strategies. The role of CGA in fall risk assessment is further discussed in the following section.

### 1.3 Fall risk assessment tool

Various assessment tools have been developed to screen patients at high risk for falls [28,29]. Frailty and sarcopenia, as significant risk factors for falls, require targeted evaluation to identify older adults at increased risk. In Japan, Toba et al. [30] developed a portable fall risk index specifically for older adults, which is validated and widely utilized in clinical settings. However, given the multifactorial nature of falls, no single global assessment tool has proven sufficient, necessitating the use of multiple tools to comprehensively evaluate risk factors [31]. One such comprehensive approach is the CGA, which integrates multiple tools to address both intrinsic and extrinsic risk factors. The CGA has been reported as useful not only for assessing fall risk but also for guiding prevention strategies. In 2024, the "Clinical and Care Guideline Based on CGA" was published in Japan, highlighting the growing importance of CGA in managing falls and related geriatric syndromes [[32], [33], [34], [35], [36]].

### 1.4 Interventions to prevent fall and fracture

Decline in physical function is a major risk factor for falls. Therefore, maintaining and improving physical function is a key component of fall prevention. However, falls cannot be attributed to physical function alone. As previously discussed, the risk of falling is multifactorial, so it is also important to consider interventions that address other intrinsic and extrinsic risk factors. From these perspectives, we describe fall prevention interventions in the following sections.

## 2 Exercise

Exercise plays a crucial role in maintaining physical function and balance, with substantial evidence supporting its benefits. Sherrington et al. [37] conducted a systematic review and found that exercise reduces fall rates by 23% compared to controls, with a pooled rate ratio of 0.77 (95% CI: 0.71–0.83). Subgroup analysis by type of exercise revealed that balance and functional exercises, as well as tai chi, were particularly effective. In addition, a study involving frail older adults reported that various exercise interventions improved frailty and enhanced physical functions, such as muscle strength and walking speed [38]. Exercise not only boosts muscle strength but also improves body composition, exercise tolerance, quality of life, and mental and neurological functions [39].

Based on these findings, the U.S. The Preventive Services Task Force recommends exercise for community-dwelling older adults aged 65 and older to prevent falls. However, in older adults, insufficient dietary intake, particularly of protein and total calories, often leads to protein energy malnutrition, which can limit functional improvements. Thus, a combined approach incorporating dietary therapies should be considered.

## 3 Nutrition

Amino acid supplementation is beneficial for muscle health. In particular, continuous supplementation of essential amino acids such as leucine and arginine is effective for improving physical functions, including increased lean body mass, lower limb strength, and walking speed [40]. Generally, the recommended protein intake for preventing sarcopenia is approximately 1.0–1.2 g/kgBW/day. In a cross-sectional study of community-dwelling older adults aged ≥ 65, we found that a low frequency of meat consumption was associated with a higher prevalence of frailty in men. Similarly, in women, a low frequency of both fish and meat consumption was linked to a higher prevalence of frailty compared with those who consumed these foods daily [41]. Additionally, weight loss, a diagnostic criterion for frailty, was strongly correlated with cooking frequency. Recent findings suggest that maintaining regular cooking habits may help prevent weight loss [42].

Taken together, despite possible gender differences, nutritional interventions are crucial for preventing frailty, sarcopenia, and falls. Ensuring adequate amino acid intake through dietary consumption, along with efforts to maintain daily cooking practices, may play a significant role in achieving these prevention goals.

## 4 Medications

As mentioned above, taking > 1 FRID is associated with an increased risk of falls; thus, discontinuing FRIDs is expected to reduce the fall risk [43]. However, due to the multifactorial nature of falls, it remains unclear whether discontinuation of FRIDs alone can decrease the incidence of falls or fall-related disability [44]. Similarly, addressing polypharmacy is crucial, as taking 5 to > 6 medications significantly increases the risk of falls, highlighting the need for careful medication adjustments [45]. Sarcopenia significantly contributes to falls, and while there are currently no approved drugs specifically for the prevention of this condition, new therapeutic options are emerging. These include testosterone, vitamin D, beta-hydroxy-beta-methyl butyrate, and traditional herbal medicines [23,[46], [47], [48]].

## 5 Bridging comprehensive assessment of fall risk to individualized intervention

Falls result from a combination of intrinsic factors, such as physical dysfunction and sensory impairments (eg, vision and hearing loss), as well as extrinsic factors, such as environmental hazards in the home. To prevent them, multifactorial interventions are needed to systematically assess modifiable risks and implement tailored strategies using CGA and other tools. In 2022, the first World Falls Guideline for Prevention and Management for Older Adults was published. The guideline outlines three core principles: (i) risk assessment and stratification; (ii) general recommendations for optimizing physical function and mobility; and (iii) a holistic, multidomain intervention for older adults at high risk of falls, with careful consideration of their priorities, beliefs, and available resources [18,49]. The inclusion of geriatrics and gerontological societies in this guideline was also a significant aspect, underscoring the importance of geriatric perspectives in fall prevention.

The CGA plays a crucial role in evaluating falls from a geriatric perspective and several studies have demonstrated the benefits in this context. A retrospective cohort study reported a significantly lower mortality rate in patients with proximal femur fractures who underwent CGA compared to those who did not, with a hazard ratio of 0.63 (95% CI: 0.45–0.87) [50]. Another study comparing the CGA group and the regular hospital management group among patients with proximal femoral fractures found that motor function, assessed four months post-surgery, was preserved in the CGA group [51]. Furthermore, CGA is reported to contribute to the correction of polypharmacy and is expected to help mitigate the fall risk associated with excessive medication use [52]. These findings strongly support the effectiveness of CGA in fracture management and highlight its potential role in fall prevention strategies. However, some studies reported that even with risk assessment and tailored interventions, falls could not be significantly reduced [53,54]. The limited effectiveness in reducing falls or fall-related injuries may be due to challenges such as protocol compliance, selection bias, or the inclusion of relatively healthy participants.

## 6 Integration of multicomponent interventions to prevent fall

Multicomponent interventions are a combination of ≥ 2 interventions from various categories, such as exercise and nutrition, delivered uniformly to all participants regardless of individual risk factors. A systematic review in people aged 60 years reported reductions in fall rate (rate ratio: 0.74, 95% CI: 0.60–0.91) and fall risk (risk ratio: 0.82, 95% CI: 0.74–0.90) [6]. In a randomized controlled trial involving patients aged 70 years with physical frailty and sarcopenia, mobility disability—defined as the inability to independently walk 400 m in less than 15 min—occurred in 46.8% of participants (283/605) in the multicomponent intervention group and 52.7% of participants (316/600) in the control group (hazard ratio: 0.78, 95% CI: 0.67–0.92; P = 0.005) among participants with short physical performance battery scores of 3–7. However, there was a higher incidence of falls in the multicomponent intervention group (80/605, 13.2%) than in the lifestyle education group (49/600, 8.2%) [55]. Given that the suggested discrepancy between self-perceived and actual physical function may lead to falls [56], the intervention may have intensified this gap. These findings highlight the importance of providing adequate, individualized guidance to ensure that interventions align with participants’ abilities and perceptions, thereby minimizing unintended risks.

## 7 Conclusions

Falls are a significant issue for older adults, making fall prevention critically important. Numerous factors contribute to falls, and comprehensive assessment and intervention using tools such as the CGA may help prevent falls and improve postfall outcomes. Although effective interventions for fall prevention in patients with frailty or sarcopenia remain unclear, exercise, nutrition, and pharmacological approaches are the central components of fall prevention strategies. Further research is needed to identify the optimal type and intensity of exercise as well as the effectiveness of combining multiple interventions to effectively prevent falls.

## CRediT author statement

**Seiji Hashimoto:** Conceptualization, Investigation, Writing–original draft, Writing–review & editing. **Tatsuya Hosoi:** Writing–review & editing, Supervision, Project administration. **Mitsutaka Yakabe:** Writing–review & editing. **Makoto Yunoki:** Writing–review & editing. **Shoya Matsumoto:** Writing–review & editing. **Yoshitaka Kase:** Writing–review & editing. **Masashi Miyawaki:** Writing–review & editing. **Masaki Ishii:** Writing–review & editing. **Sumito Ogawa:** Writing–review & editing, Supervision, Funding acquisition.

## Conflicts of interest

Sumito Ogawa received lecture fees from 10.13039/501100022274Daiichi Sankyo (Japan), 10.13039/501100012351Mitsubishi Tanabe Pharma (Japan), and 10.13039/501100013420Tsumura (Japan). S. Ogawa received research funding from 10.13039/501100013420Tsumura (Japan). The other authors declare no competing interests.

## References

[1] Burns E.R., Stevens J.A., Lee R. (2016). The direct costs of fatal and non-fatal falls among older adults - United States. J Saf Res.

[2] World Health Organization (2021).

[3] The prevention of falls in later life (1987). A report of the Kellogg International Work Group on the Prevention of Falls by the Elderly. Dan Med Bull.

[4] Verma S.K., Willetts J.L., Corns H.L., Marucci-Wellman H.R., Lombardi D.A., Courtney T.K. (2016). Falls and fall-related injuries among community-dwelling adults in the United States. PLoS One.

[5] Wang J., Chen Z., Song Y. (2010). Falls in aged people of the Chinese mainland: epidemiology, risk factors and clinical strategies. Ageing Res Rev.

[6] Hopewell S., Adedire O., Copsey B.J., Boniface G.J., Sherrington C., Clemson L. (2018). Multifactorial and multiple component interventions for preventing falls in older people living in the community. Cochrane Database Syst Rev.

[7] Dautzenberg L., Beglinger S., Tsokani S., Zevgiti S., Raijmann R.C.M.A., Rodondi N. (2021). Interventions for preventing falls and fall-related fractures in community-dwelling older adults: a systematic review and network meta-analysis. J Am Geriatr Soc.

[8] Mirelman A., Rochester L., Maidan I., Del Din S., Alcock L., Nieuwhof F. (2016). Addition of a non-immersive virtual reality component to treadmill training to reduce fall risk in older adults (V-TIME): a randomised controlled trial. Lancet.

[9] Moreland B., Kakara R., Henry A. (2020). Trends in nonfatal falls and fall-related injuries among adults aged ≥65 years - United States, 2012-2018. MMWR Morb Mortal Wkly Rep.

[10] Hartholt K.A., Lee R., Burns E.R., van Beeck E.F. (2019). Mortality from falls among US adults aged 75 years or older, 2000-2016. JAMA.

[11] de Vries M., Seppala L.J., Daams J.G., van de Glind E.M.M., Masud T., van der Velde N. (2018). Fall-risk-increasing drugs: a systematic review and meta-analysis: I. Cardiovascular drugs. J Am Med Dir Assoc.

[12] Seppala L.J., Wermelink A.M.A.T., de Vries M., Ploegmakers K.J., van de Glind E.M.M., Daams J.G. (2018). Fall-risk-increasing drugs: a systematic review and meta-analysis: II. Psychotropics. J Am Med Dir Assoc.

[13] Seppala L.J., van de Glind E.M.M., Daams J.G., Ploegmakers K.J., de Vries M., Wermelink A.M.A.T. (2018). Fall-risk-increasing drugs: a systematic review and meta-analysis: III. Others. J Am Med Dir Assoc.

[14] Matsumoto S., Tamiya H., Yamana H., Hosoi T., Matsui H., Fushimi K. (2023). Association between the type of hypnotic drug and in-hospital fractures in older patients with neurocognitive disorders: a case-control study using a nationwide database. Geriatr Gerontol Int.

[15] Moreland B.L., Kakara R., Haddad Y.K., Shakya I., Bergen G. (2021). A descriptive analysis of location of older adult falls that resulted in emergency department visits in the United States, 2015. Am J Lifestyle Med.

[16] Cauley J.A., Hovey K.M., Stone K.L., Andrews C.A., Barbour K.E., Hale L. (2019). Characteristics of self-reported sleep and the risk of falls and fractures: the Women’s Health Initiative (WHI). J Bone Miner Res.

[17] Espinosa R., Ponce H., Gutiérrez S., Martínez-Villaseñor L., Brieva J., Moya-Albor E. (2019). A vision-based approach for fall detection using multiple cameras and convolutional neural networks: a case study using the UP-Fall detection dataset. Comput Biol Med.

[18] Seppala L.J., van der Velde N. (2022). How to tackle the global challenge of falls?. Age Ageing.

[19] Colón-Emeric C.S., McDermott C.L., Lee D.S., Berry S.D. (2024). Risk assessment and prevention of falls in older community-dwelling adults: a review. JAMA.

[20] Yang Z.C., Lin H., Jiang G.H., Chu Y.H., Gao J.H., Tong Z.J. (2023). Frailty is a risk factor for falls in older adults: a systematic review and meta-analysis. J Nutr Health Aging.

[21] Kinoshita K., Satake S., Matsui Y., Arai H. (2021). Association between sarcopenia and fall risk according to the muscle mass adjustment method in Japanese older outpatients. J Nutr Health Aging.

[22] Xue Q.L. (2011). The frailty syndrome: definition and natural history. Clin Geriatr Med.

[23] Guo X., Pei J., Ma Y., Cui Y., Guo J., Wei Y. (2023). Cognitive frailty as a predictor of future falls in older adults: a systematic review and meta-analysis. J Am Med Dir Assoc.

[24] Shen S., Xie Y., Zeng X., Chen L., Guan H., Yang Y. (2023). Associations of intrinsic capacity, fall risk and frailty in old inpatients. Front Public Health.

[25] Hosoi T., Yakabe M., Hashimoto S., Akishita M., Ogawa S. (2024). The roles of sex hormones in the pathophysiology of age-related sarcopenia and frailty. Reprod Med Biol.

[26] Chen W.C., Li Y.T., Tung T.H., Chen C., Tsai C.Y. (2021). The relationship between falling and fear of falling among community-dwelling elderly. Medicine (Baltim).

[27] Pilotto A., Custodero C., Maggi S., Polidori M.C., Veronese N., Ferrucci L. (2020). A multidimensional approach to frailty in older people. Ageing Res Rev.

[28] Podsiadlo D., Richardson S. (1991). The timed “Up & Go”: a test of basic functional mobility for frail elderly persons. J Am Geriatr Soc.

[29] Tinetti M.E. (1986). Performance-oriented assessment of mobility problems in elderly patients. J Am Geriatr Soc.

[30] Toba K., Okochi J., Takahashi T., Matsubayashi K., Nishinaga M., Yamada S. (2005). Development of a portable fall risk index for elderly people living in the community. Jpn J Geriatr.

[31] Strini V., Schiavolin R., Prendin A. (2021). Fall risk assessment scales: a systematic literature review. Nurs Rep.

[32] Hosoi T., Ogawa S., Shibasaki K., Akishita M. (2025). Special issue: comprehensive geriatric assessment (CGA)-based healthcare guidelines 2024. Geriatr Gerontol Int.

[33] Efendioglu E.M., Cigiloglu A., Ozturk Z.A. (2023). The role of comprehensive geriatric assessment in predicting fall risk. Ir J Med Sci.

[34] Xiao X., Li L., Yang H., Peng L., Guo C., Cui W. (2023). Analysis of the incidence of falls and related factors in elderly patients based on comprehensive geriatric assessment. Aging Med (Milton).

[35] Magnuszewski L., Wojszel A., Kasiukiewicz A., Wojszel Z.B. (2022). Falls at the geriatric hospital ward in the context of risk factors of falling detected in a comprehensive geriatric assessment. Int J Environ Res Publ Health.

[36] Tricco A.C., Thomas S.M., Veroniki A.A., Hamid J.S., Cogo E., Strifler L. (2017). Comparisons of interventions for preventing falls in older adults: a systematic review and meta-analysis. JAMA.

[37] Sherrington C., Fairhall N., Kwok W., Wallbank G., Tiedemann A., Michaleff Z.A. (2020). Evidence on physical activity and falls prevention for people aged 65+ years: systematic review to inform the WHO guidelines on physical activity and sedentary behaviour. Int J Behav Nutr Phys Activ.

[38] Yang X., Li S., Xu L., Liu H., Li Y., Song X. (2024). Effects of multicomponent exercise on frailty status and physical function in frail older adults: a meta-analysis and systematic review. Exp Gerontol.

[39] Joseph S.M., Rich M.W. (2017). Targeting frailty in heart failure. Curr Treat Options Cardiovasc Med.

[40] Borsheim E., Bui Q.U., Tissier S., Kobayashi H., Ferrando A.A., Wolfe R.R. (2008). Effect of amino acid supplementation on muscle mass, strength and physical function in elderly. Clin Nutr.

[41] Shibasaki K., Kin S.K., Yamada S., Akishita M., Ogawa S. (2019). Sex-related differences in the association between frailty and dietary consumption in Japanese older people: a cross-sectional study. BMC Geriatr.

[42] Hoshi K., Shibasaki K., Yakabe M., Hosoi T., Matsumoto S., Yamada S. (2025). Relationship between decreased activities of daily living, decreased physical strength and future weight loss in community-dwelling older adults. Geriatr Gerontol Int.

[43] Zia A., Kamaruzzaman S.B., Tan M.P. (2017). The consumption of two or more fall risk-increasing drugs rather than polypharmacy is associated with falls. Geriatr Gerontol Int.

[44] Lee J., Negm A., Peters R., Wong E.K.C., Holbrook A. (2021). Deprescribing fall-risk increasing drugs (FRIDs) for the prevention of falls and fall-related complications: a systematic review and meta-analysis. BMJ Open.

[45] Kojima T., Akishita M., Nakamura T., Nomura K., Ogawa S., Iijima K. (2012). Polypharmacy as a risk for fall occurrence in geriatric outpatients. Geriatr Gerontol Int.

[46] Rolland Y., Dray C., Vellas B., Barreto P.S. (2023). Current and investigational medications for the treatment of sarcopenia. Metabolism.

[47] Yakabe M., Ogawa S., Ota H., Iijima K., Eto M., Ouchi Y. (2018). Inhibition of interleukin-6 decreases atrogene expression and ameliorates tail suspension-induced skeletal muscle atrophy. PLoS One.

[48] Yakabe M., Hosoi T., Sasakawa H., Akishita M., Ogawa S. (2022). Kampo formula hochu-ekki-to (Bu-Zhong-Yi-Qi-Tang, TJ-41) ameliorates muscle atrophy by modulating atrogenes and AMPK in vivo and in vitro. BMC Complement Med Ther.

[49] Montero-Odasso M., van der Velde N., Martin F.C., Petrovic M., Tan M.P., Ryg J. (2022). World guidelines for falls prevention and management for older adults: a global initiative. Age Ageing.

[50] Pajulammi H.M., Pihlajamäki H.K., Luukkaala T.H., Jousmäki J.J., Jokipii P.H., Nuotio M.S. (2017). The effect of an in-hospital comprehensive geriatric assessment on short-term mortality during orthogeriatric hip fracture program - which patients benefit the most?. Geriatr Orthop Surg Rehabil.

[51] Prestmo A., Hagen G., Sletvold O., Helbostad J.L., Thingstad P., Taraldsen K. (2015). Comprehensive geriatric care for patients with hip fractures: a prospective, randomised, controlled trial. Lancet.

[52] Hosoi T., Yamana H., Tamiya H., Matsui H., Fushimi K., Akishita M. (2022). Association between comprehensive geriatric assessment and polypharmacy at discharge in patients with ischaemic stroke: a nationwide, retrospective, cohort study. EClinicalMedicine.

[53] Bhasin S., Gill T.M., Reuben D.B., Latham N.K., Ganz D.A., Greene E.J. (2020). A randomized trial of a multifactorial strategy to prevent serious fall injuries. N Engl J Med.

[54] Lamb S.E., Bruce J., Hossain A., Ji C., Longo R., Lall R. (2020). Screening and intervention to prevent falls and fractures in older people. N Engl J Med.

[55] Bernabei R., Landi F., Calvani R., Cesari M., Del Signore S., Anker S.D. (2022). Multicomponent intervention to prevent mobility disability in frail older adults: randomised controlled trial (SPRINTT project). BMJ.

[56] Hayashi S., Misu Y., Sakamoto T., Yamamoto T. (2023). Cross-sectional analysis of fall-related factors with a focus on fall prevention self-efficacy and self-cognition of physical performance among community-dwelling older adults. Geriatrics (Basel).

